# Eclampsia

**Published:** 1905-05

**Authors:** Frances Bradley


ATLANTA

### Journal-Record of Medicine.

**Successor to Atlanta Medical and Surgical Journal, Established 1855, and Southern Medical Record, Established 1870.**

OWNED BY THE ATLANTA MEDICAL JOURNAL CO.

**Published Monthly.**

VOL. VII. MAY, 1905. No. 2.

BERNARD WOLFF, M.D., EDITOR, Nos. 319-20 Prudential.

M. B. HUTCHINS, M.D., BUSINESS MANAGER, 1007-1008 Century Bldg.

J. N. LECONTE, M.D., ASSOCIATE EDITOR.

### *ORIGINAL COMMUNICATIONS.*

### ECLAMPSIA.

BY FRANCES BRADLEY, M.D.

One of the most interesting complications of the pregnant, parturient or puerperal state, is that of eclampsia; interesting to the patient, for it endangers not only her own life but that of her child; interesting to the physician, for its very elusiveness, calling forth his most alert attention, his most careful, conscientious efforts, and opening up a field for valuable original work.

*Frequency:* Eclampsia occurs most often in the latter half of pregnancy, less often during labor, and rarely in the puerperium. It occurs most often in primiparae or in cases of multiple pregnancy where the tension of the abdominal wall is at its greatest. It occurs oftener in hospital than in private practice, such cases properly finding their way to those institutions. Williams reports 1-133 cases from hospital records as opposed to 1-500 for private work. There is an unaccountable difference in statistics of different years, which might be more or less suggestive to the men of the micro-organism theory.

For instance, Tarnier's Clinic at Paris reports one case eclampsia for every 47 labors in 1872; one case eclampsia for every 730 labors in 1882; one case eclampsia for every 130 labors in 1892. No explanation of this diversity of statistics has yet been offered.

*Etiology:* Perhaps the best proof of the uncertain character of our knowledge and the experimental nature of our work on eclampsia lies in the evolution of the present-day theory as to its etiology. Early writers considered it a neurosis, due probably to the hydremia of pregnancy and its effect on an overwrought and unstable central nervous system. It was later considered identical with uremia till the two conditions were demonstrated to have little in common. Anemia and edema of the brain had also to be abandoned in view of the fact that the post-mortem findings failed often to bear out one's expectations. Then came the idea of bacterial invasion, only to give way to what seemed the more rational theory of autointoxication. Several observers, among them Tarnier, of Paris, and Bouffe de Saint Blaise, demonstrated the existence of a decrease in the toxicity of urine with a corresponding increase in the toxicity of the blood serum. Even the investigations of such men proved disappointing, and we are reduced to the working premise that eclampsia is caused by the circulation in the blood of some toxic substance which gives rise to thrombosis in many of the smaller vessels, especially in the liver, kidneys and brain, causing degenerative and necrotic changes in those organs. Whether these changes are due to a fibrin ferment, to an excess of globulin, to products of fetal metabolism which in certain cases overwhelm the maternal organism, it is impossible to say. It has been generally observed that the convulsions cease with the birth or death of the fetus. And in many cases the same liver and kidney lesions have been found in mother and child dying at or soon after eclamptic seizure.

There are certain predisposing causes which one can not afford to ignore in any case of pregnancy:

1. Conditions which predispose to hepatic insufficiency.

2. Any acute or chronic kidney lesion, though it be nothing more than the German "kidney of pregnancy."

3. Retention of urine from any cause, pathological or mechanical, as

(a) Abnormal enlargement of uterus, hydremia, multiple pregnancy, etc.

(b) Small pelvis or large head.

(c) Rigid muscles—primiparae, especially if very young or old.

As exciting causes one must recognize the most trivial irritant, external or internal, and it is most important for the eclamptic woman to be kept relaxed and quiet, absolutely free from strain, mental or physical.

*Pathology:* Ewing, who has spent the past seven years in a careful study of the toxemia of pregnancy, considers nausea and vomiting, ictanus and eclampsia as manifestations of the same condition, due to the absorption of an excess of toxic material, which in the pregnant state the overcharged organs are unable to eliminate. These vary in character and intensity with the resistance of the woman and the extent of the intoxication.

He reports from repeated autopsies the most constant and characteristic changes in the liver. Schmorl finds in seventeen autopsies seventeen defective livers. Bouffe de Saint Blaise finds in forty-two consecutive post-mortems as many livers showing degenerative or necrotic areas surrounding a thrombotic portal vein. Usually this change is evident even to the naked eye. Always he finds more characteristic changes in the liver than in any other organ.

Williams, of Johns Hopkins, confirms these findings, and adds that he has found such lesions in no other condition.

The kidneys also are often the seat of degenerative or necrotic changes, sometimes showing simply the swollen albuminuric kidney of pregnancy, at others a pregnancy complicated by an acute or even a chronic lesion. But in view of the number of cases reported showing marked liver changes with slight or no renal insufficiency, it behooves us to hold ourselves in a receptive, or better, an investigative attitude, and be prepared to readjust our ideas on the albuminuria of pregnancy, and the relation of nephritis to eclampsia.

In addition to liver and kidney changes are found similar lesions in the brain, necrotic areas surrounding thrombi in the smaller cerebral vessels.

*Symptoms:* Certain prodromal symptoms will usually attract the attention of the alert physician. I would beware of the woman who becomes restless and irritable, of the woman with a puffy eye, of one who complains of dull, persistent frontal headache with dizziness, of epigastric pain and indigestion, of general debility, and above all, of one who shows any inactivity of the liver or kidneys. The presence of albumen, of any decrease in total amount of urine or in percentage of urea, marks her as a case of faulty metabolism, and unless elimination can be promptly established, one has a right to expect the later and classic symptoms of the eclamptic seizure with which we are all familiar.

*Diagnosis:* Failing to anticipate trouble or seeing a patient for the first time in convulsions, one must differentiate between uremia epilepsy, hysteria and eclampsia. In the uremic woman there would possibly be urinous breath. There would be marked edema of face and extremities. The dyspnea of the uremic woman is said to be more pronounced than that of the eclamptic. There would be partial or total suppression of urine in the uremic patient, with the classic symptoms of a nephritis acute or chronic, none of which are necessarily present in the woman of the eclamptic convulsions. The hysterical patient will show no edema, will do herself no violence, may laugh or cry, may be roused from her stupor, her temperature and urine may be normal. In the epileptic patient one can usually get a history of previous attacks of a premonitory aura in the present one. There will be no edema, no alteration in urinary or hepatic function, slight if any rise in temperature, and a dilated pupil if seen early in the attack.

*Prognosis:* The prognosis is always grave, 20-30 per cent. maternal, while that of the child runs up to 33-50 per cent. The prognosis is better the longer one can stave off attacks, the longer the interval between them. It improves in proportion to the responsiveness of the excretory organs to treatment, the liver, kidneys, bowels, skin and lungs. It improves if a hard, wiry pulse can be converted into a soft, compressible one. Lastly it improves with the birth or death of the fetus.

*Treatment:* The treatment of eclampsia is necessarily experimental in consequence of our uncertain knowledge as to its etiology. The only point upon which all authorities agree, is that the most compensating if less brilliant treatment lies in the prevention rather than the cure of the condition; in the early recognition of the toxemic tendency and the skillful guidance of our patient past the shoals of toxic manifestations. With this end in view the *first symptom* should excite one's prompt and careful attention. The patient should be put on a fruit and liquid diet, kept out of doors as much as possible, urged to drink lavishly of Lithia and Carlsbad, take a hot bath and a copious hot drink each night before retiring. This gentle stimulus to the excretory organs will often serve to correct a beginning trouble. If not, I should give the patient a hot pack followed by a pill containing one grain each of calomel, digitalis and squills, and 1-20 grain muriate of pilocarpine, unless contraindicated by a bad heart. The excretory organs will rarely fail to respond to this treatment and in twenty-four hours to show a marked improvement in symptoms. Given a convulsion, one has two patients instead of one to consider, and prompt action modified by good judgment is doubly important. First, I would protect the cheeks and tongue from ugly bites by wedging a twisted towel or sheet between the teeth.

In the interest of the child, uterine contractions must be controlled, and for this purpose chloroform is perhaps the sheet-anchor, though chloral hydrate by the rectum and morphine hypodermetically may answer the same purpose. The convulsive seizure past, the question arises shall we treat the case symptomatically or regard the symptoms as manifestations of a condition which must be removed before relief can be expected. There is usually a hard, wiry pulse indicative of the tense heart action. A few years ago this was met by veratrum viride, or a similar depressant. Edgar still recommends its use in full doses, and I believe it finds favor in this part of the country. But Williams of Johns Hopkins finds other drugs more efficacious, and the Boston men, headed by those of Massachusetts General Hospital, after its careful and continuous use, have discarded it as a dangerous depressant on an already overworked heart. Personally, I have had what seemed to me good results from its use, a pulse hard and rapid becoming soft, compressible and slow. Further than this I can not go. And I must say that pilocarpine, while not safe in all cases, will yet give the same action on the heart with added diaphoresis, which I failed to get with veratrum. Stimulation of kidneys and skin may be reinforced by large, high enemas of salt solution, and the same may be used subcutaneously. Calomel, or a drop of croton oil in zi olive oil well back on the tongue will clear out the gastrointestinal tract. Convulsion failing to abate under these precautions, I should feel that the welfare of the child, if viable, demanded delivery, as soon as compatible with the safety of the mother. Just here it is important to remember that the eclamptic woman, already the victim of profound intoxication, is painfully susceptible to infection, and that surgical cleanliness is imperative at every step.

If the external os is the only obstacle to delivery, I should dilate digitally and deliver by version or forceps, according as the head is high or low. The cervix still intact, I should dilate with a glove stretcher or pack with gauze till at least the internal os has disappeared. If in a hospital or aided by a trained assistant, one of the hydrostatic dilators, preferably that of Champetier de Ribbes, approaches more nearly the natural process of dilation.

Other measures may be necessary in rare and complicated cases. But I have tried to confine myself to the commoner forms which we must all be prepared to meet. What I have said is prompted not so much by what I know of eclampsi, as by what I want to know, and I shall be very grateful for any suggestions, or for a free discussion of the subject.

---

### WOMAN DOCTOR APPOINTED PRIVATDOCENT IN SWITZERLAND.

Madame Schwuenter, M.D., has recently been admitted to the position of Privatdocent in Dermatology in the University of Berne. She is the first woman to hold such a position there.

